# Cutaneous Pediatric Vasculitis: A Clinico-Histopathological Overview of Common and Novel Entities

**DOI:** 10.3390/dermatopathology13030037

**Published:** 2026-08-18

**Authors:** Anne Welfringer-Morin, Stéphanie Leclerc-Mercier

**Affiliations:** 1 Department of Dermatology, Hôpital Necker-Enfants Malades, Assistance Publique—Hôpitaux de Paris, 75015 Paris, France; 2 Department of Pathology, Hôpital Necker-Enfants Malades, Assistance Publique—Hôpitaux de Paris, 75015 Paris, France

**Keywords:** pediatrics, vasculitis, histology, monogenic

## Abstract

Vasculitis encompasses a heterogeneous group of diseases characterized by the inflammation of blood vessels. In children, the diagnosis of vasculitis with cutaneous involvement relies on a combination of clinical evaluation, histopathological examination, and, in some cases, genetic investigations. Diagnosing pediatric vasculitis remains particularly challenging due to overlapping clinical manifestations, variable disease courses, and the evolving nature of histological features. Histopathological assessment aims to confirm inflammation of the vessel walls—either readily visible at low magnification or requiring serial sections—and to characterize the inflammatory infiltrate and other key features that aid in classifying the vasculitis subtype. Accurate diagnosis requires ongoing dialog and collaboration among multiple specialists. In this article, we present the clinical and histopathological features of the most common pediatric vasculitides—including IgA vasculitis and polyarteritis nodosa—as well as newly recognized entities such as COVID-19-associated vasculitis and monogenic vasculitides.

## Simple Summary

Pediatric vasculitis comprises a heterogeneous group of disorders marked by blood vessel inflammation, with its diagnosis based on clinical, histopathological, and sometimes genetic evaluation. It is challenging to diagnose due to overlapping features and variable presentations, making histopathology key for confirming and classifying the disease. Accurate diagnosis requires multidisciplinary collaboration, and this article reviews common and emerging forms, including IgA vasculitis, polyarteritis nodosa, and newer entities such as COVID-19-associated and monogenic vasculitides.

## 1. Introduction

Vasculitis is a heterogeneous group of diseases characterized by the inflammation of blood vessels. The Chapel Hill classification proposes a nomenclature based on the size of the affected vessels [1]. In this classification, cutaneous involvement corresponds to small vessels. A revised nomenclature by a group of dermatologists [2] has expanded the group of cutaneous vasculitis to include the involvement of small- and medium-sized vessels.

Cutaneous vasculitis encompasses several entities:

- Cutaneous component of a systemic vasculitis;
- Skin-limited or skin-dominant expression or variant of a systemic vasculitis;
- Single-organ vasculitis.

Vasculitis represents a complex group that can involve multiple organs, leading to significant morbidity and mortality. A classification of pediatric vasculitis [3], considering the specificities of children, has been proposed, based on the size of the involved vessels and the presence of granulomas. This classification allows for optimized management, follow-up, and targeted treatment of the pediatric population.

The epidemiology of pediatric vasculitis is scarcely reported, with incidence in the UK estimated at 23–50 per 100,000 inhabitants with variable distribution among ethnic groups [2].

In this article, we detail the clinical and histopathological aspects of the most common pediatric vasculitis (IgA vasculitis, Kawasaki disease, and PAN) as well as newly recognized entities such as COVID-19-induced vasculitis and monogenic vasculitis.

## 2. Who Diagnoses Pediatric Vasculitis?

The diagnosis of pediatric vasculitis with cutaneous involvement relies on a combination of clinical, histological, and sometimes genetic findings. Diagnosing pediatric vasculitis remains challenging due to overlapping clinical features, variability in disease progression, and the dynamic nature of histopathological findings.

Dermatologists base their approach on:

- The size of the vessels: livedo = large; purpura = small; nodule = deep vessel.
- The presence of systemic symptoms such as neuropathy, alveolar hemorrhage, arthralgia, myalgia.

The histological diagnosis aims to identify the inflammatory involvement of the vessel walls, which may be evident at low magnification or require serial cuts, and to analyze the nature of the inflammatory infiltrate and specific features that help classify the vasculitis.

Accurate diagnosis requires continuous dialog and collaboration among multiple specialists. The complexity and clinical diversity of monogenic vasculitis often necessitate genetic confirmation in order to optimize patient management and therapeutic strategies.

## 3. Common Pediatric Vasculitis

### 3.1. IgA Vasculitis

IgA vasculitis (IgAV), formerly named Henoch–Schönlein purpura, is the most common pediatric vasculitis. It is a small-vessel vasculitis characterized by IgA-dominant immune deposits. IgAV accounts for approximately 50% of pediatric vasculitis cases, with a higher incidence in males compared to other types of vasculitis. The incidence reported is 3–26 per 100,000 children [4]. The disease predominantly affects children between 5 and 15 years of age, with a male-to-female ratio of approximately 2:1.

**Figure 1:**
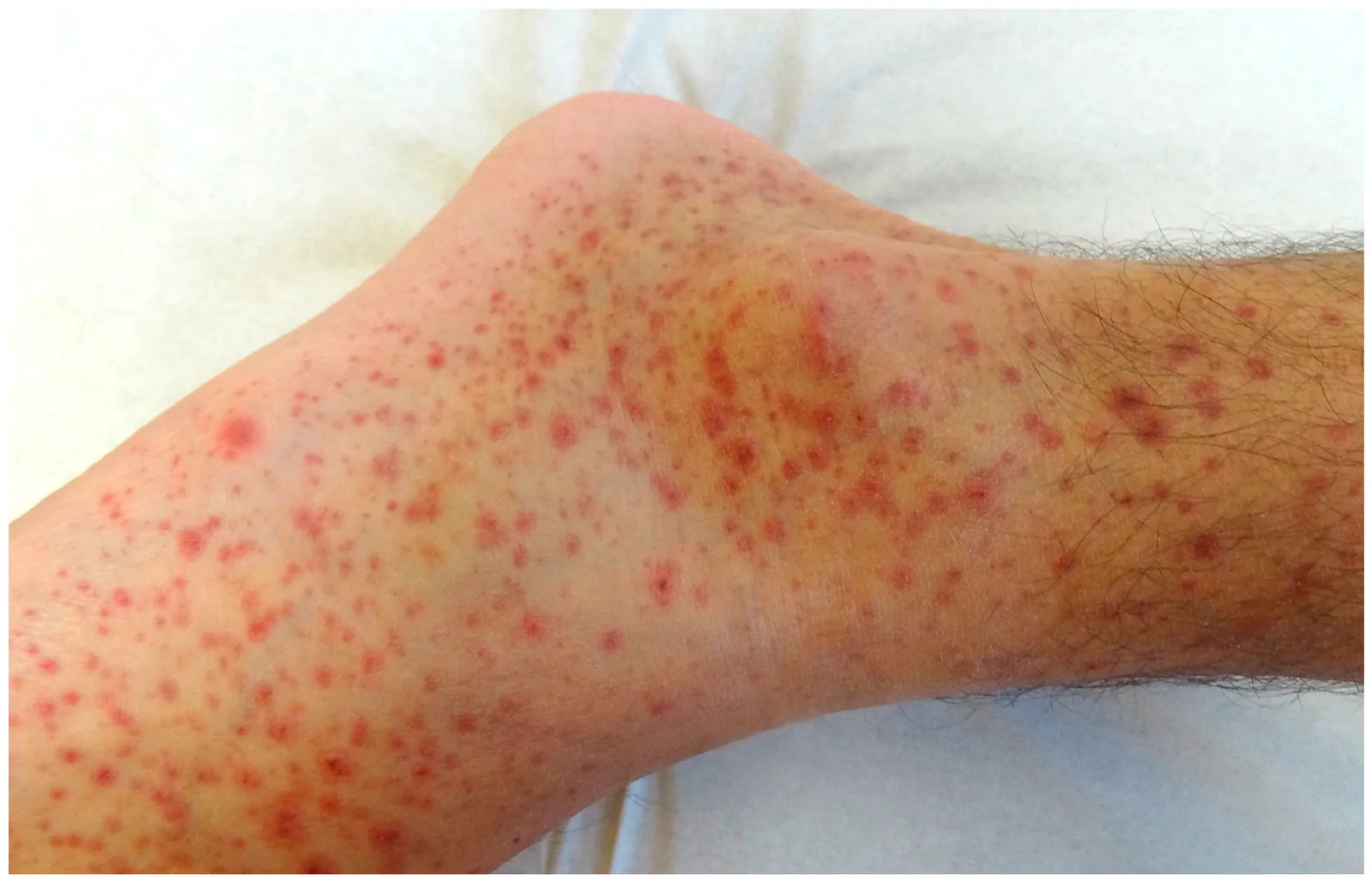
IgA vasculitis, palpable purpura with crusted and necrotic lesions. This figure is from Necker dermatology department and illustrates the corresponding clinical symptoms.

Upper respiratory tract infections play a significant role in the onset of IgAV, with a clear seasonal pattern characterized by peaks during the fall and winter months. Clinically, pediatric IgAV commonly presents with a purpuric rash predominantly located on the buttocks and lower limbs [3]. The skin involvement is present in almost 100% of cases and often precedes other clinical manifestations. The rash evolves in flare-ups, with lesions at different stages of development. It presents as a palpable purpura, which may be necrotic, bullous, or crusted (Figure 1). The purpura is symmetrical, preferentially located at pressure points and in dependent areas [5]. Subcutaneous edema is common, likely secondary to increased capillary permeability. Arthritis occurs in approximately 75% of cases, with 15% presenting with joint symptoms as the initial manifestation. Gastrointestinal manifestations affect 30 to 50% of patients. Diffuse abdominal pain is the most frequent symptom and may be associated with vomiting, rectal bleeding, or diarrhea. Rare digestive complications may occur, including gastrointestinal bleeding, acute intestinal intussusception, pancreatitis, and exudative enteropathy [6].

Renal involvement of IgAV occurs in 20–80% of children and can present with isolated microscopic (and/or macroscopic) haematuria with or without proteinuria, nephritic and/or nephrotic syndrome [7]. Renal involvement should therefore be systematically assessed at diagnosis and throughout follow-up, with blood pressure measurement and urine dipstick testing performed weekly during the first two months, then monthly for one year [7].

Other complications may occur, including orchitis, neurological involvement, or pulmonary involvement.

Early recognition and appropriate management of IgAV are crucial to mitigate potential complications, particularly renal impairment, which can have long-term consequences if not addressed promptly.

The classification criteria from EULAR/PRINTO/PRES [3] are:

- Typical purpura and at least one of the following criteria:
  ○ Abdominal pain;
  ○ Arthritis or arthralgia;
  ○ Renal involvement (proteinuria or hematuria);
  ○ Typical histology (leukocytoclastic vasculitis or proliferative glomerulonephritis with IgA deposits).

**Figure 2:**
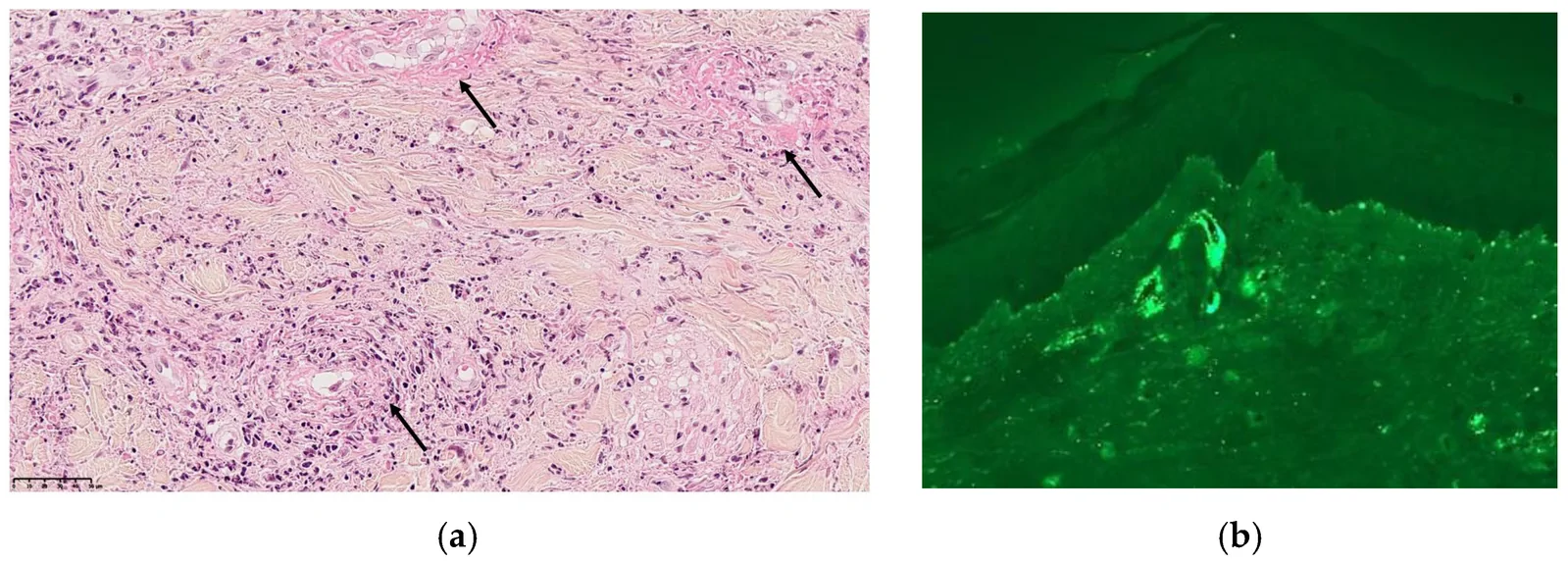
(**a**). IgA vasculitis, skin biopsy, hematein eosin, ×40 magnification. Fibrinoid necrosis of vessel walls (arrows), accompanied by neutrophilic infiltration around and within the vessel walls, leukocytoclasia; (**b**). direct immunofluorescence demonstrating IgA deposition within the vessel walls. These figures are from Necker Pathology department and illustrate the corresponding pathology.

Skin biopsy (standard histology and immunofluorescence) is not mandatory for diagnosis and should be reserved for atypical rashes or when a differential diagnosis needs to be excluded. If performed, a skin biopsy should be taken from the edge of a recent purpuric lesion to optimize histopathological analysis. Histologically, IgA vasculitis is a leukocytoclastic vasculitis, often showing fibrinoid necrosis of vessel walls, with neutrophilic infiltration around and within the vessel walls, and sometimes, marked leukocytoclasia (Figure 2a). Extravasation of red blood cells is often observed. Eosinophils are also frequently present [8,9].

Direct immunofluorescence (DIF) studies typically reveal IgA deposits, which can be associated with IgG and C3 deposits [10] (Figure 2a). The 1990 American College of Rheumatology (ACR) classification [11] was based solely on histological features, whereas the 2008 Ankara classification [3] specified IgA deposits as a diagnostic criterion. The presence of IgA has a sensitivity of 100% and a specificity of 87% in the pediatric population. Their sensitivity and specificity in the adult population with IgAV have also shown very favorable values (99.2% and 86%, respectively), which support their use, regardless of age [12].

In 2019, a European consensus was published [7], providing recommendations on performing skin and renal biopsies. Skin biopsy and DIF are not systematically required but are recommended in cases of atypical rash or when the exclusion of differential diagnoses is necessary. Notably, the absence of IgA deposits does not rule out the diagnosis of IgA vasculitis (IgAV).

For direct immunofluorescence, a biopsy should be obtained at the edge of a fresh purpuric lesion (<24–48 h old), preferably on the lower extremities.

The therapeutic management relies on analgesics (use of anti-inflammatory drugs as first-line treatment, except in cases of renal involvement or gastrointestinal bleeding) and bed rest for skin improvement. Systemic corticosteroid therapy may be considered in the event of complications such as orchitis, severe gastrointestinal involvement, or neurologic involvement. In cases of glomerulonephritis, treatment decisions should be made in collaboration with a pediatric nephrologist. IgAV is most often a benign condition with spontaneous resolution, but renal monitoring (blood pressure measurement and urine dipstick testing) should therefore continue for at least 6 to 12 months, even when the initial findings are normal.

### 3.2. IgA Vasculitis Variant/Acute Hemorrhagic Edema of Infancy

Acute hemorrhagic edema is a skin-limited small-vessel leukocytoclastic vasculitis that typically occurs following a mild febrile illness, most often of viral origin. It primarily affects infants under 3 years old, with a median age of 11 months [13], and is not observed in adults. This condition is confined to the skin, follows a benign course, and resolves itself spontaneously. It is considered an infantile variant of IgA vasculitis.

**Figure 3:**
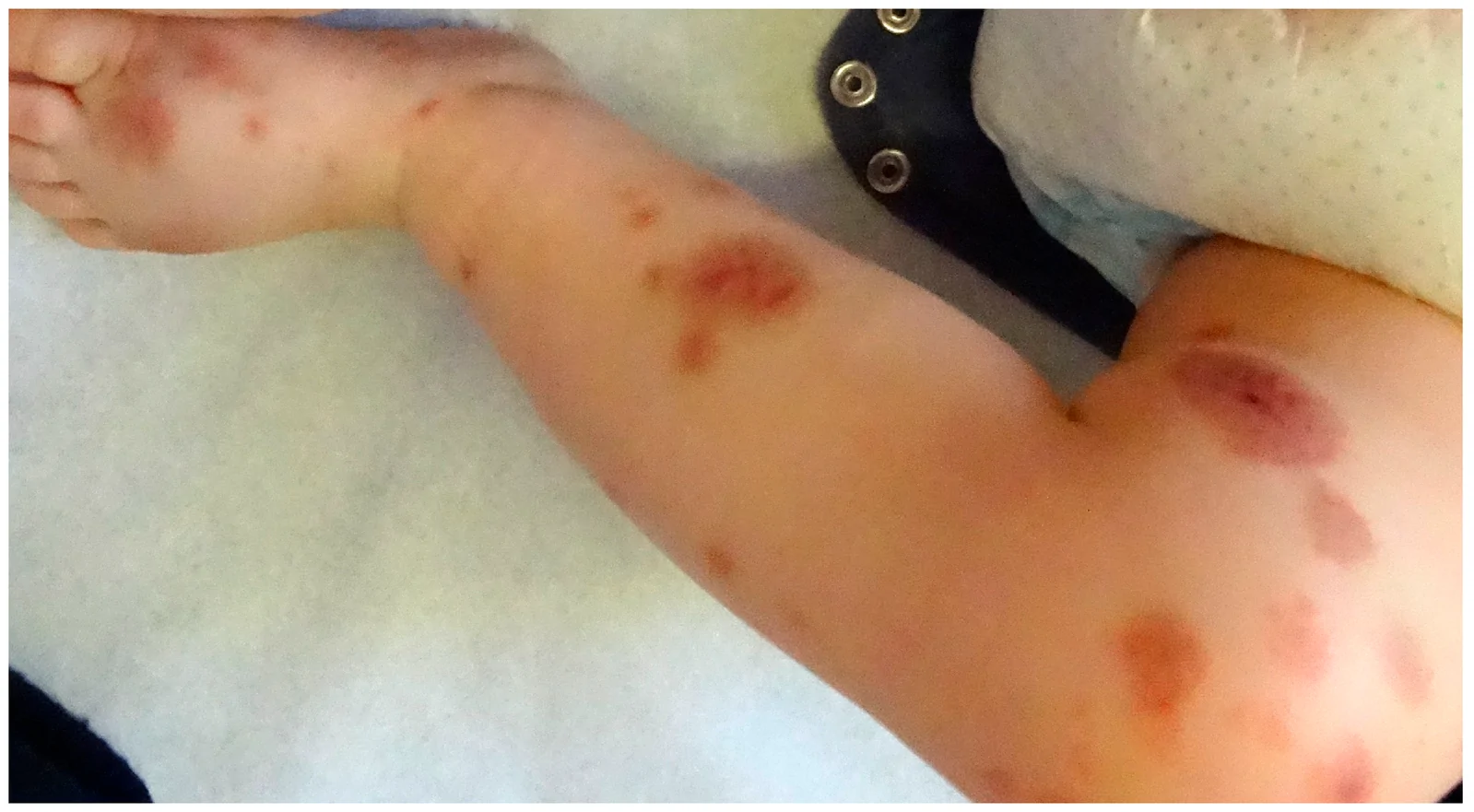
Acute Hemorrhagic Edema of Infancy, purpuric lesions in the leg with ecchymotic and annular lesions. This figure is from Necker dermatology department and illustrates the corresponding clinical symptoms.

It is characterized by palpable purpuric lesions, predominantly affecting the face, ears, arms, forearms, thighs, and legs [14]. The lesions are often associated with hemorrhagic edema and present as ecchymotic, annular, or nummular plaques that are symmetrically distributed and bilateral. Fever is frequently present. Pruritus is absent (Figure 3).

Children generally remain in good overall condition.

**Figure 4:**
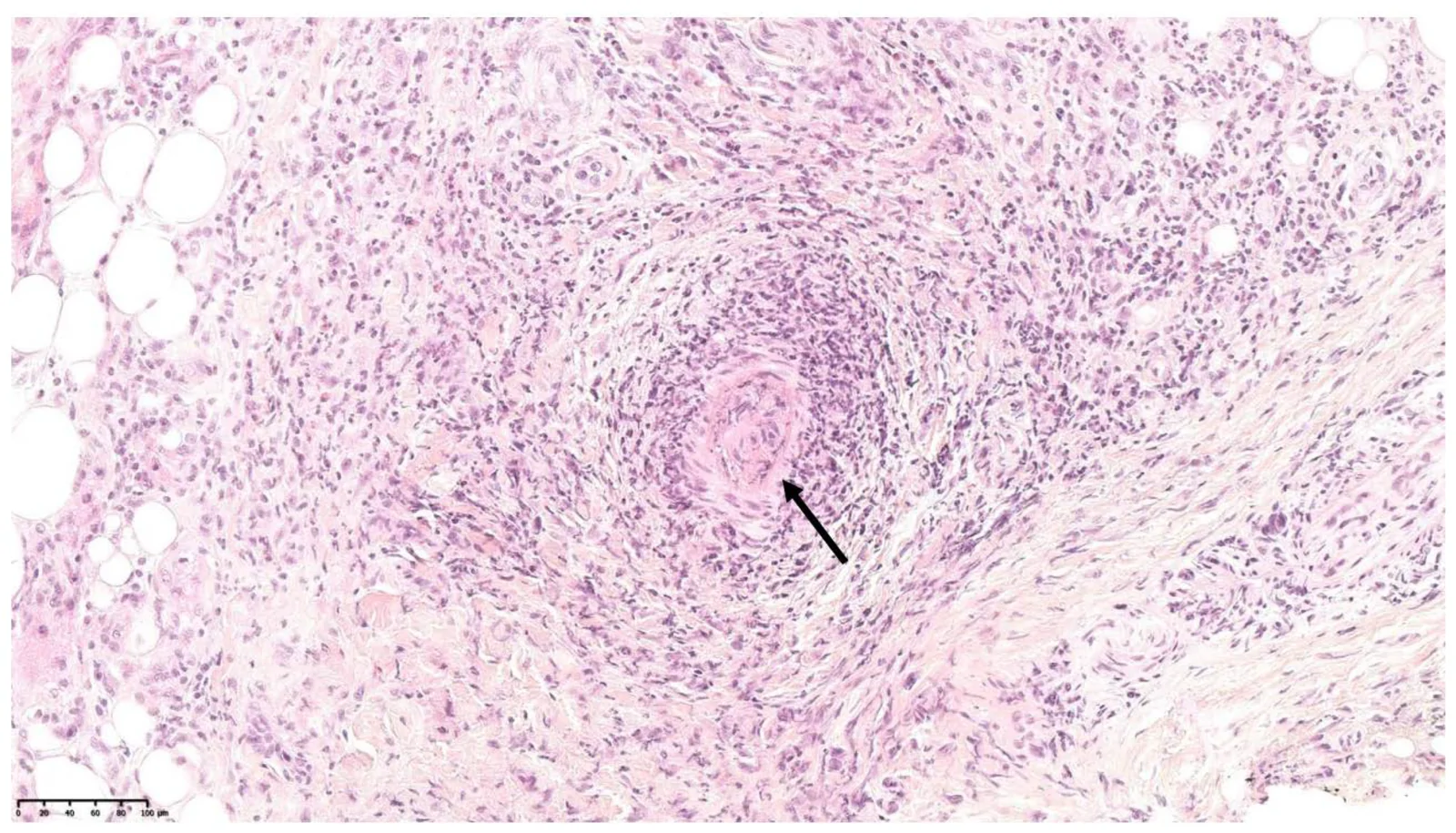
Acute Hemorrhagic Edema of Infancy, skin biopsy, hematein eosin, ×20 magnification. Neutrophilic infiltrate around a deep vessel, leukocytoclasia, and fibrinoid necrosis (arrow). This figure is from Necker Pathology department and illustrates the corresponding pathology.

A skin biopsy is rarely required, except in atypical cases, as the clinical presentation is usually distinctive enough to establish the diagnosis. Histological findings are infrequently reported in the literature but resemble those of IgAV, with a neutrophilic infiltrate around and within small vessels, leukocytoclasia, and often fibrinoid necrosis. Distinctive features include the inflammatory infiltrate, which is more polymorphous, comprising lymphocytes, neutrophils, and eosinophils, with a characteristic distribution that is perivascular and also deep, peri-adnexal, and interstitial [15,16] (Figure 4).

The course of acute hemorrhagic edema is typically self-limited, with spontaneous resolution occurring within approximately ten days. No specific treatment is required other than antipyretics. Skin lesions resolve completely without sequelae and do not require any targeted intervention.

## 4. Polyarteritis Nodosa

Limited epidemiological data are available on pediatric polyarteritis nodosa (PAN); however, it is the third most common vasculitis in children, after IgAV and Kawasaki disease, and the second most frequent when considering cutaneous involvement. It is a necrotizing vasculitis that affects small- and medium-sized vessels. Unlike in adults, PAN in children is not associated with Hepatitis B infection.

In systemic PAN, clinical manifestations include fever, skin involvement, arthralgia, and myalgia, as well as gastrointestinal, neurologic, and renal involvement, and are typically accompanied by markedly elevated inflammatory markers (increased C-reactive protein and neutrophil count). Cutaneous involvement is observed in 20% to 60% of systemic PAN cases and includes subcutaneous nodules, livedo, purpura, and skin ulcers. Isolated cutaneous forms of PAN have also been described. The diagnosis is confirmed by biopsy [17].

Antineutrophil cytoplasmic antibodies (ANCA) are typically negative in PAN.

**Figure 5:**
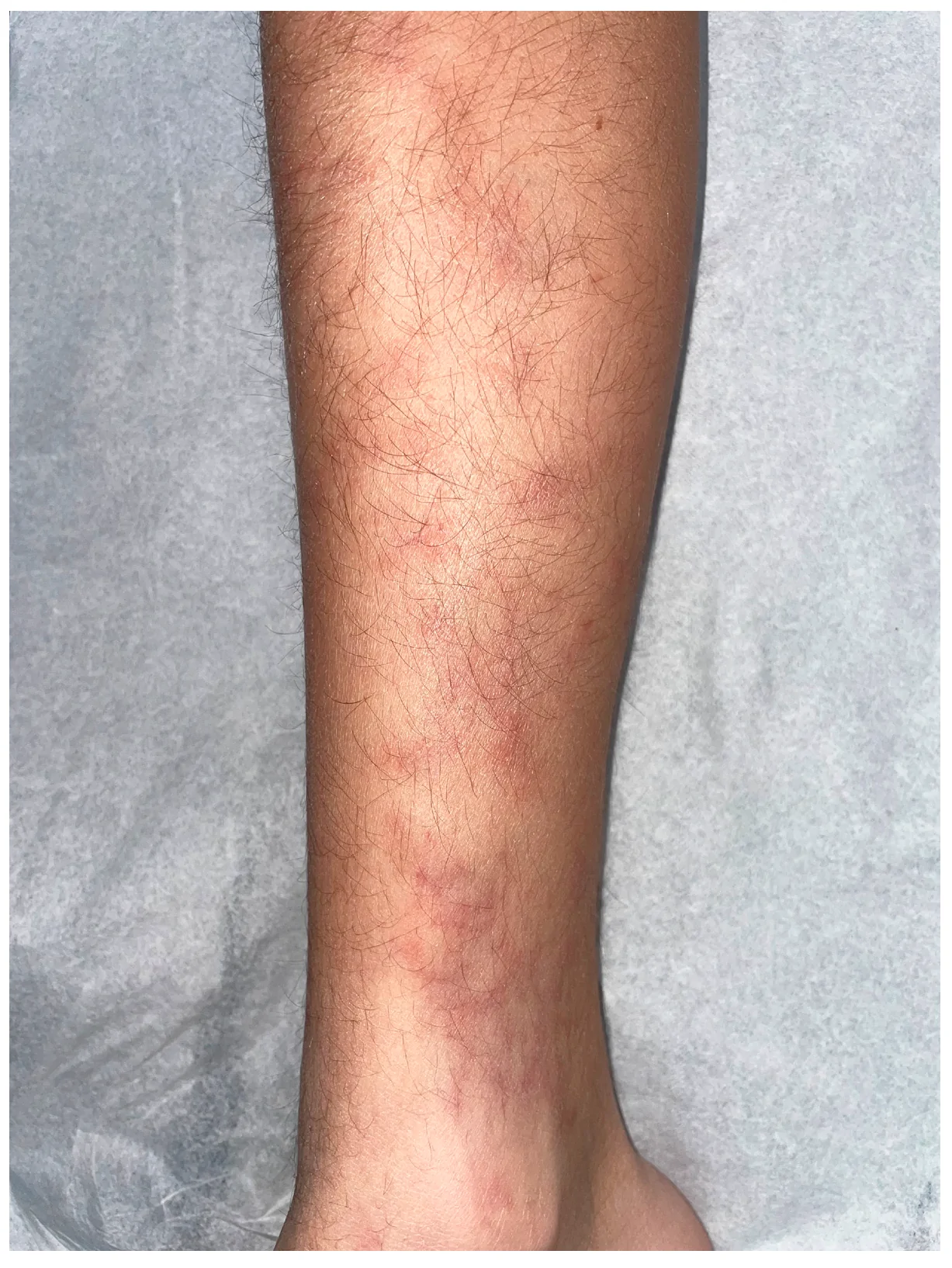
PAN, livedo, branched, with large open meshes. This figure is from Necker dermatology department and illustrates the corresponding clinical symptoms.

The disease usually presents at around 9 to 10 years of age [18], with skin and neuromuscular involvement being the most common features. Dermatological pediatric presentations often include nodules and livedo (Figure 5).

Systemic PAN relapses more frequently in children.

The diagnostic criteria for PAN were revised in the 2008 Ankara classification, making histological abnormalities a mandatory criterion for diagnosis in children [3].

The histological diagnosis of PAN is significantly enhanced through detailed and precise communication with the clinician. For patients with suspected cutaneous involvement of PAN, performing a deep skin biopsy is recommended—extending to the medium-sized vessels in the deep dermis and the subcutis—rather than a superficial punch biopsy, as it may provide more diagnostic value [19].

Serial cuts are required since the vascular involvement is segmental and focal.

**Figure 6:**
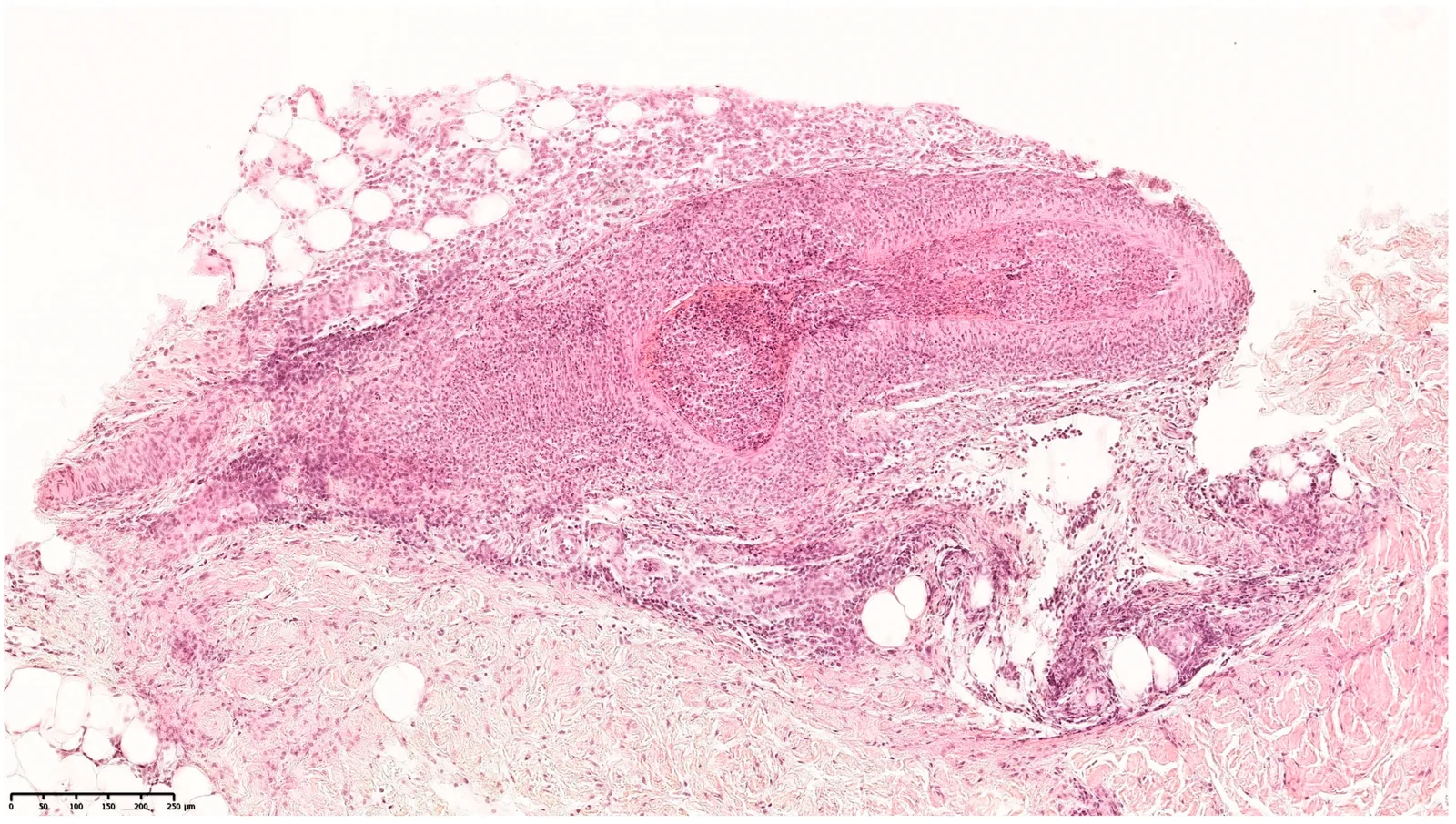
PAN, skin biopsy, hematein eosin, ×10 magnification. Vasculitis of a medium-sized vessel in the deep dermis, with a dense perivascular infiltrate composed of neutrophils. This figure is from Necker Pathology department and illustrates the corresponding pathology.

**Figure 7:**
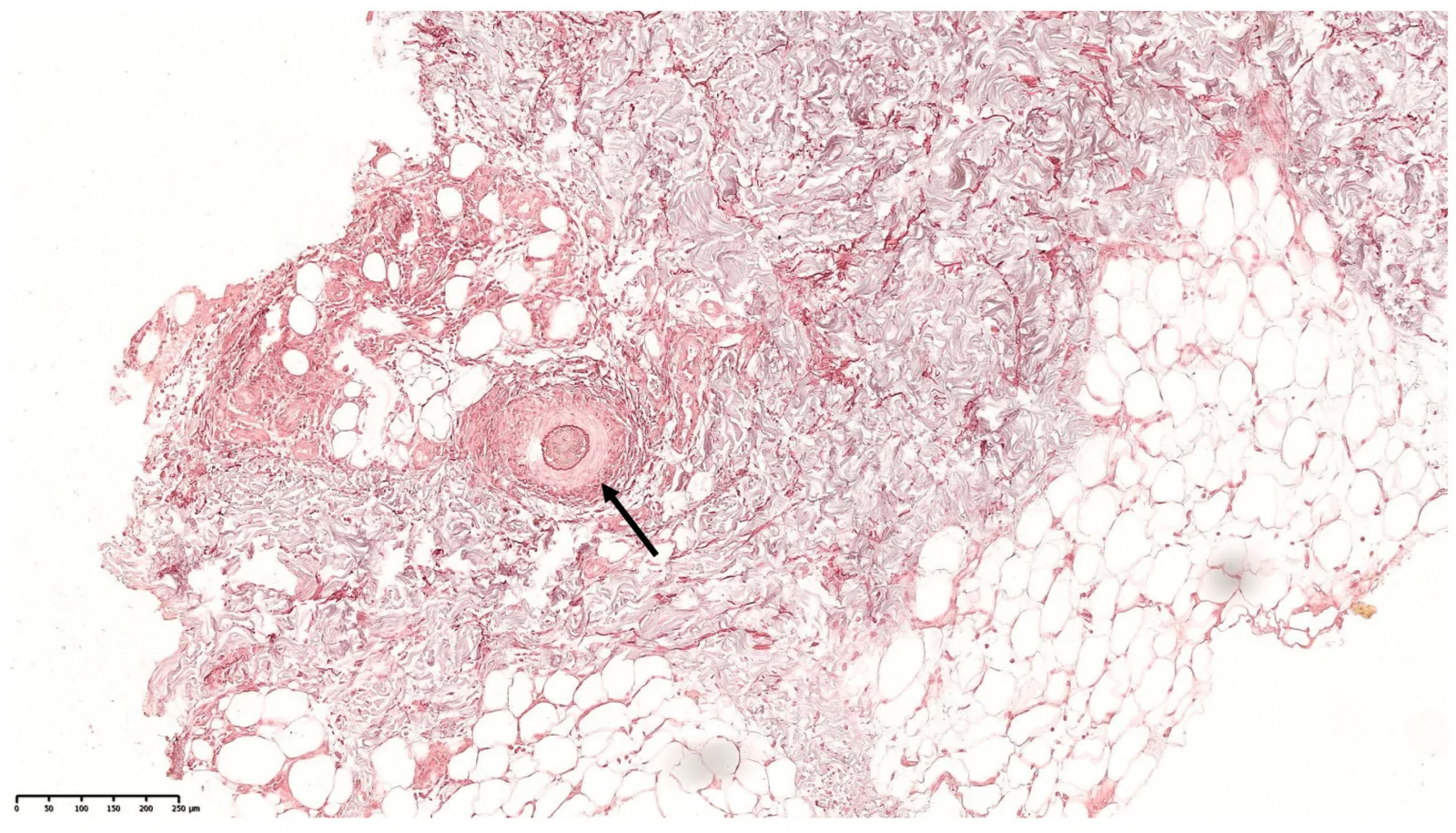
PAN, skin biopsy, orcein staining, ×10 magnification. Orcein staining confirms the arterial nature of the vessel by revealing an internal elastic lamina (arrow). Notably, on sections performed after hematoxylin-eosin staining, the inflammatory infiltrate is no longer visible, supporting the segmental and focal nature of the lesion. This figure is from Necker Pathology department and illustrates the corresponding pathology.

The diagnosis of cutaneous PAN is based on the presence of histopathological features of necrotizing vasculitis of medium-sized arteries, including fibrinoid necrosis, leukocytoclasia, neutrophilic infiltration around and within arterial walls, and extravasation of red blood cells. Eosinophils and lymphocytes can be present. Different stages of development can be observed. The inflammation is localized around the artery, which differentiates it from that of panniculitis [9,20] (Figure 6 and Figure 7).

These findings are crucial for confirming the diagnosis of PAN.

Sometimes, leukocytoclastic vasculitis of the superficial dermal vessels is also observed [9].

Isolated cutaneous forms of PAN are treated with NSAIDs and corticosteroids, combined with close clinical and biological monitoring. In the case of systemic forms, treatment in an expert center should be discussed (corticosteroid therapy, immunosuppressive therapy). Overall survival is better, and the risk of relapse is lower in children than in adults [18].

## 5. Kawasaki Disease

Kawasaki disease is primarily a pediatric vasculitis of the medium and, to a lesser degree, small vessels. It has a particular tropism for coronary arteries and typically affects children under 5 years of age. It is the second most common systemic vasculitis in children, after IgA vasculitis.

Kawasaki disease is considered an autoinflammatory condition. The severity of this vasculitis is due to its selective affinity for the coronary arteries, which can lead to aneurysms in 25% of untreated children. Unlike other types of vasculitis, Kawasaki disease does not present with vasculitis-like skin involvement.

Diagnosis [21], as defined by the American Heart Association (AHA), is established in a child with a sudden onset of fever persisting for more than 5 days, unresponsive to antipyretics, and accompanied by at least four of the following criteria:

- Changes in extremities: erythema and palmoplantaredema during the acute phase, and desquamation of the extremities and/or perineum during the subacute phase.
- Cervical lymphadenopathy greater than 1.5 cm in diameter.
- Rash: non-specific maculopapular erythema
- Alterations in lips and oral mucosa: cheilitis, glossitis (strawberry tongue), or oropharyngitis.
- Bilateral non-purulent conjunctivitis.

Cutaneous manifestations of Kawasaki disease are part of the clinical diagnostic criteria; however, skin biopsies are not specific without vasculitis. A Skin biopsy is not recommended as a diagnostic criterion by the European consensus or the French National Diagnostic and Care Protocol, given its lack of specificity in Kawasaki disease, and is almost never performed [22,23]. A biopsy of skin eruption was reported as psoriasiform epidermal aspect with an erythema multiforme-like interface dermatitis [24].

Sugawara reports significant edema accompanied by dilation of small vessels within the papillary dermis. Neutrophil infiltration remains minimal, with the majority of inflammatory cells being mononuclear [25].

## 6. New Entities

### COVID-19-Induced Vasculitis

The COVID-19 pandemic has been associated with skin manifestations directly linked to the infection and, in some cases, to vaccination. In children, manifestations associated with histological vasculitis, primarily affecting small vessels, mainly include chilblain-like lesions, livedoid and purpuric lesions, and IgA vasculitis [26] during the first peak of the COVID-19 pandemic.

Finally, an entity known as Pediatric Multisystem Inflammatory Syndrome (PIMS) temporally associated with SARS-CoV-2 (PIMS-TS), which has been compared to Kawasaki disease, was described during this period. It includes systemic vasculitis, rash (52%), myocarditis (66% of cases), multisystem involvement, and hypercoagulation. The mean age of patients with PIMS is 7.9 years old [27].

Concerning skin manifestations, a polymorphic rash has been reported in 20% to 70% of cases. Perineal and facial desquamation, as well as abnormalities of the hands and feet (including erythema, edema, and induration), have also been described.

Since cutaneous lesions can result from both the direct viral effect and the immune response triggered by the infection, histological findings may reflect either viral cytopathic changes or secondary inflammatory processes [27]. Skin histology is not mandatory and not specific.

**Figure 8:**
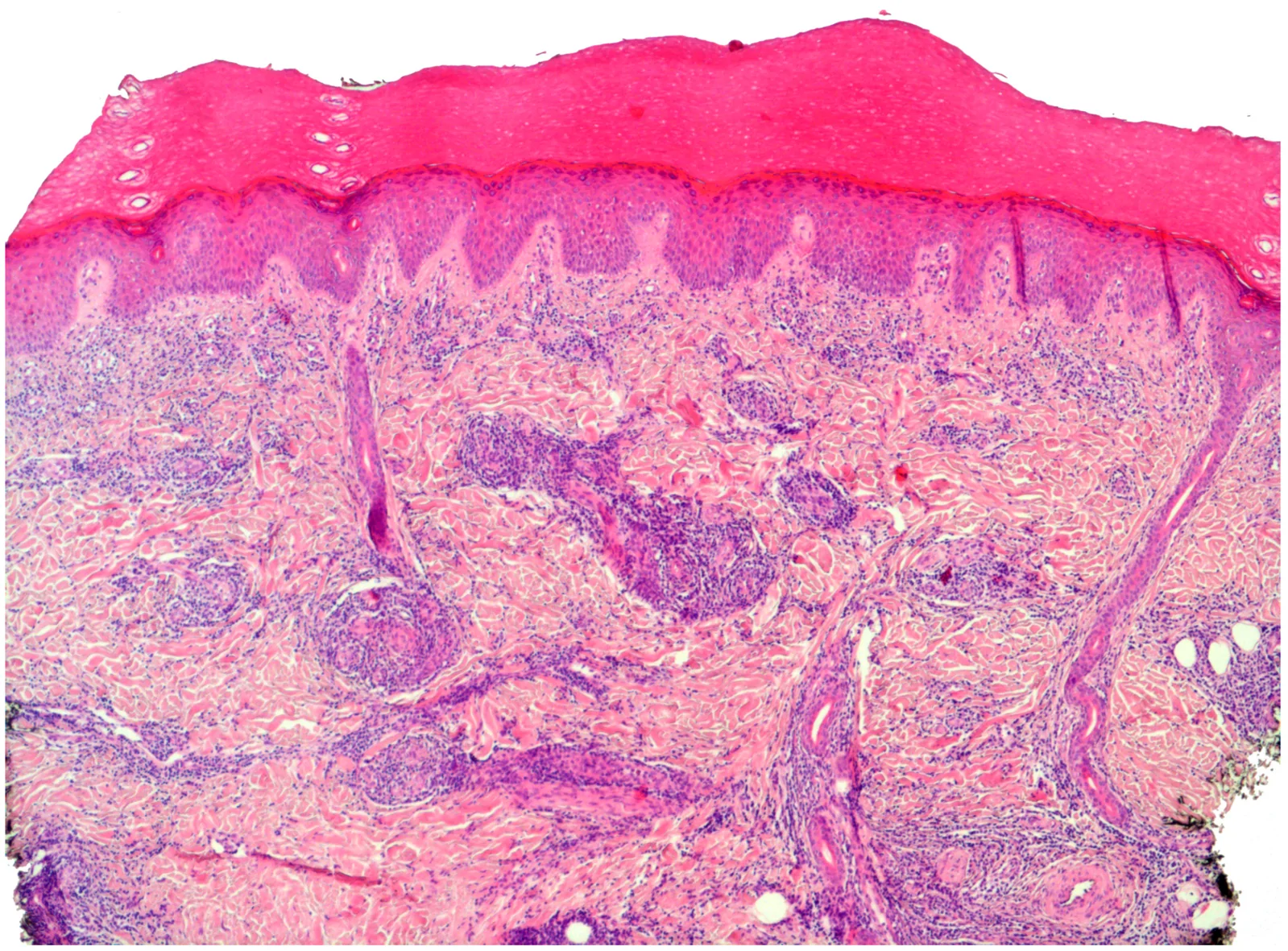
COVID-19 chilblains, skin biopsy, hematein eosin, ×10 magnification. Intense lymphocytic vasculitis of small vessels (superficial + deep dermis). The figure is from Prof. A. Kolivras’ collection and illustrates the corresponding pathology.

Chilblain-like lesions show lymphocytic vasculitis of small vessels (superficial + deep dermis) with variably fibrinoid necrosis, endothelitis, thrombosis, and extravasation of red blood cells [26]. Histopathologically, COVID-19-induced chilblains are indistinguishable from those caused by other primary or secondary factors [28] (Figure 8).

In these lesions, the SARS-CoV-2 spike protein has been detected in endothelial cells. In livedoid and purpuric lesions, thrombosis of small dermal vessels is observed, with minimal inflammatory infiltrate.

Direct immunofluorescence (DIF) studies have reported deposits of IgM, C3, and fibrinogen in the vessel walls, along with C5b-9 and C4d. Again, the SARS-CoV-2 spike protein has been detected in endothelial cells.

## 7. Monogenic Vasculitis

### 7.1. Deficiency of Adenosine Deaminase 2 (DADA2)

Deficiency of adenosine deaminase 2 (DADA2) is an autosomal recessive autoinflammatory disease caused by loss-of-function mutations in the ADA2 gene (previously the *CECR1* gene) on chromosome 22q11.1. First described in 2014 [29,30], >600 cases have been reported.

Patients with DADA2 have several features related to autoinflammatory syndromes: recurrent fever, aphtous ulcers, myalgia, and arthralgia/arthritis.

Disease manifestations could be separated into three major phenotypes: inflammatory/vascular, immune dysregulatory (hypogammagloubulinemia and cytopenia), and lymphoproliferation (hepatosplenomegaly and lymphadenopathie). Clinically, cutaneous manifestations are the most common: livedo reticularis, cutaneous features resembling polyarteritis nodosa, Raynaud’s phenomenon, and ulcers or necrosis.

Neurological involvement, such as stroke, is frequent, affecting half of the cases.

The vascular involvement in DADA2 can extend to multiple other organs. A history of periodic, non-infectious fever is frequently present [31].

Erythrocyte sedimentation rate (71%) and C-reactive protein (84%) were elevated.

The disease typically manifests early, with 24% of cases presenting before one year of age and 77% before ten years. Mortality occurs in 8% of cases before the age of 30, primarily due to strokes or infections.

DADA2 is a vasculitis affecting small- and medium-sized arteries, which can be the initial manifestation of the disease.

In cases presenting with livedo, skin biopsies are close to the PAN aspect and typically reveal interstitial infiltration by neutrophils around and within small- and medium-sized arteries, and macrophages. For pseudo-PAN nodules, histopathological examination often shows necrotizing vasculitis characterized by fibrinoid necrosis [30,31,32,33,34]. Later studies have described non-specific leukocytoclastic vasculitis and panniculitis in skin biopsy findings.

**Figure 9:**
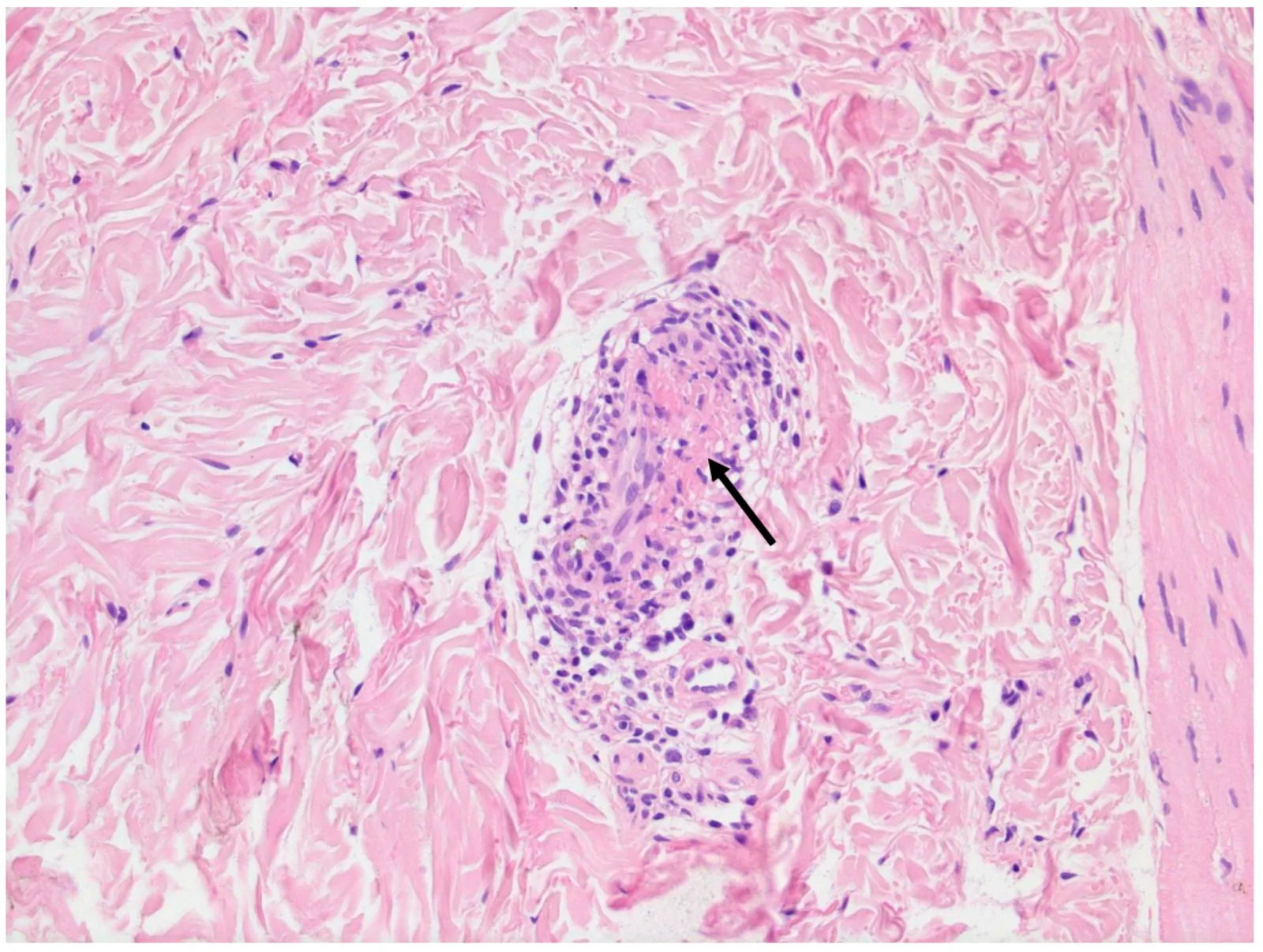
DADA2, skin biopsy, hematein eosin, ×40 magnification. Small-vessel lymphocytic vasculitis with fibrinoid necrosis (arrow). The figure is from Prof. A. Kolivras’ collection and illustrates the corresponding pathology.

Kolivras et al. also reported a case with a mononuclear cell infiltrate surrounding thrombosed intermediate-sized vessels simulating lymphocytic thrombophilic arteritis [34] (Figure 9).

Early recognition and genetic testing are crucial for appropriate management and genetic counseling. The treatment of DADA2 depends on the clinical phenotype and disease severity. Treatment strategies may include anti-tumor necrosis factor (TNF) therapy in vasculitis/vasculopathy and inflammatory phenotypes of DADA2, which has shown efficacy in preventing ischemic events. In patients with severe hematologic abnormalities or immunodeficiency, hematopoietic stem cell transplantation (HSCT) may be considered.

Anticoagulation and aspirin are currently not recommended by the NIH due to concerns about the risk of hemorrhagic stroke.

### 7.2. STING-Associated Vasculopathy with Onset in Infancy (SAVI)

Activating mutations in *STING1* were described in 2014 to cause a novel type I interferonopathy, referred to as SAVI [35,36]. This disease is typically characterized by early-onset systemic inflammation with fever, skin vasculopathy, joint inflammation, and interstitial lung disease [37].

Cutaneous lesions predominantly affect facial areas (cheeks, nose, ears) and distal extremities (hands, feet), presenting as telangiectasias, chilblain-like lesions, livedo, violaceous nodules or plaques, and ulcerations. Perforation of the nasal septum is recorded [37,38]. Nail involvement includes dystrophy and, in severe cases, gangrene of fingers or toes. Alopecia is possible. Nailfold capillaroscopy often reveals no specific microangiopathy with tortuous capillaries.

Pulmonary manifestations include interstitial lung disease and pulmonary fibrosis. Articular manifestations are frequent. Biologically, there is an inflammatory syndrome with increased levels of C-reactive protein and/or erythrocyte sedimentation rate. The IFN score and levels of IFNα protein were consistently high in all patients tested.

Early recognition and diagnosis of SAVI are crucial for appropriate management and improving patient outcomes.

**Figure 10:**
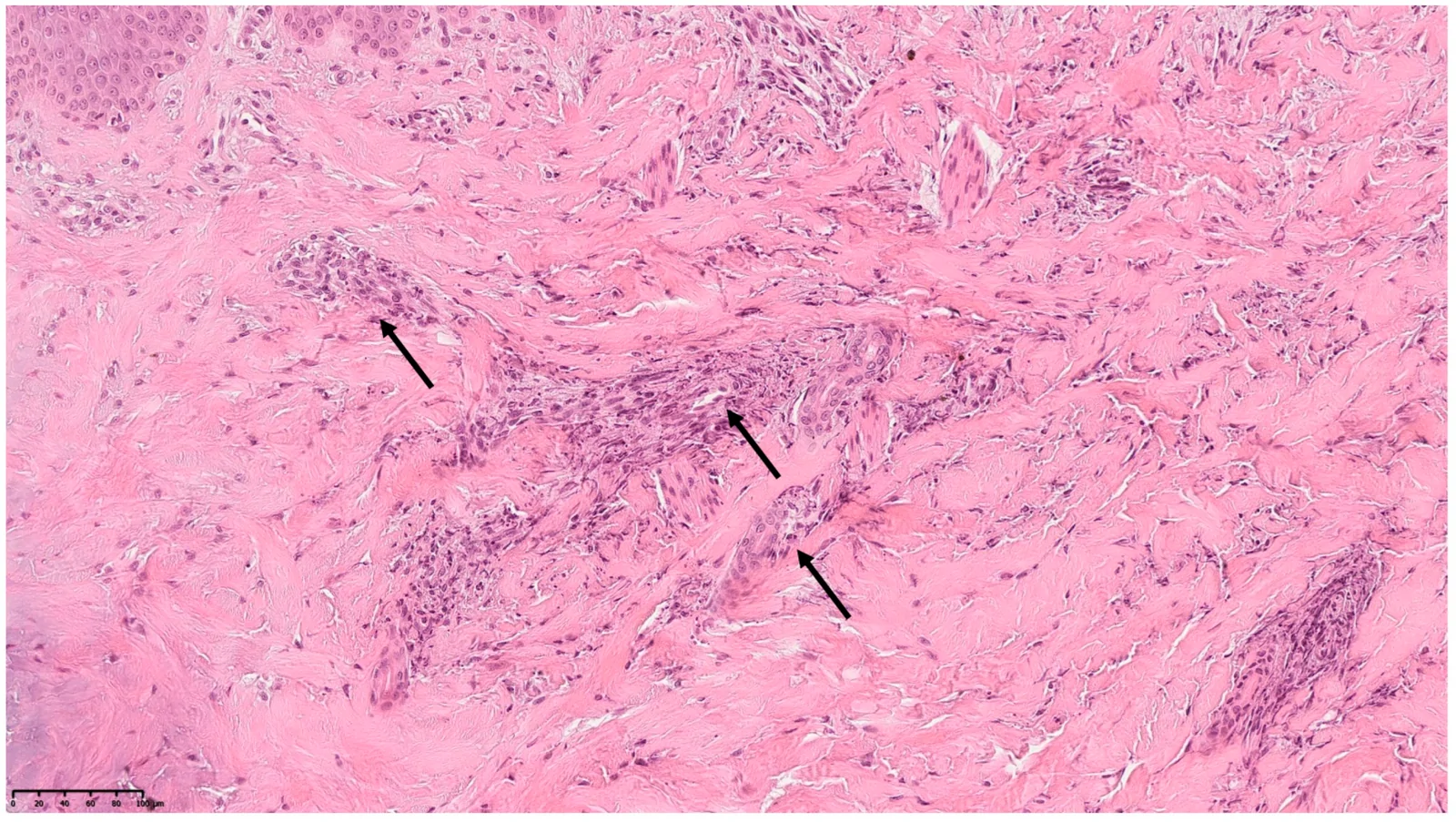
SAVI, vasculopathic lesions (arrows), necrotic chilblains. This figure is from Necker Pathology department and illustrates the corresponding pathology.

**Figure 11:**
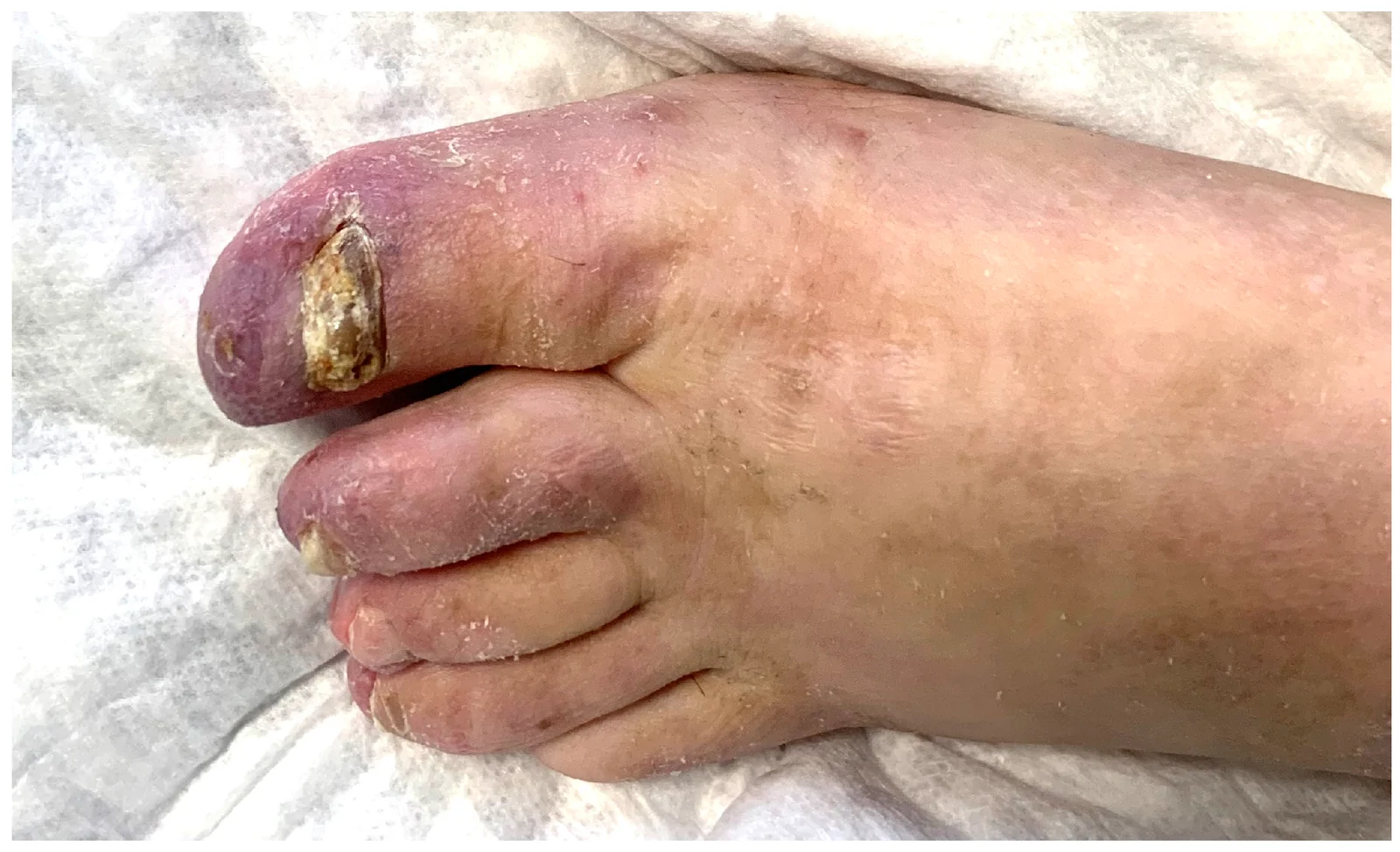
SAVI, skin biopsy, hematein eosin, ×20 magnification. Small-vessel lymphocytic vasculitis and neutrophilic infiltrate. This figure is from Necker dermatology department and illustrates the corresponding clinical symptoms.

Performing a skin biopsy (Figure 10) in safely accessible lesional areas can be useful for identifying thrombotic microangiopathy, small-vessel lymphocytic vasculitis and sometimes, neutrophilic dermatosis [34,35,39] (Figure 11).

This severe autoinflammatory condition was minimally responsive to conventional immunosuppressive therapies and thus was associated with marked childhood morbidity and increased mortality. JAK inhibitors allow rapid resolution of fever, with variable improvement in pulmonary and cutaneous manifestations depending on disease severity and the extent of tissue damage [35,40].

### 7.3. Other Auto-Inflammatory Diseases Associated with Vasculitis

Auto-inflammatory diseases can present with different histologic patterns including a vasculitic pattern [34].

Small-sized vessels can present with leukocytoclastic vasculitis [familial Mediterranean fever (FMF), Pyrin-associated auto-inflammation with neutrophilic dermatosis (PAAND), neonatal onset multisystem inflammatory disorder (NOMID), PAPA (pyogenic arthritis, pyoderma gangrenosum, acne), and hyperimmunoglobulinemia D syndrome]. Cutaneous lesions have been reported as morbilliform, urticarial, and purpuric rashes. Auto-inflammatory disease can present a thrombotic microangiopathy (particularly in interferonopathies, Aicardi–Gouttiere syndrome, familial chilblain lupus) or lymphocytic vasculitis [TNF receptor-associated periodic syndrome (TRAPS)]. The thrombotic microangiopathy in the interferonopathies causes severe necrotic chilblains, leading to digital amputations and tissue loss. In TRAPS syndrome, periorbital edema and cellulitis-like subcutaneous inflammation of the upper limbs are characteristic feature.

Intermediate-sized vessels are affected in DADA-2 (see above).

## 8. Other Types of Vasculitis Less Frequent in Children

### ANCA-Related Vasculitis

ANCA-related types of vasculitis include granulomatous polyangiitis (GPA), microscopic polyangiitis (MPA), Wegener’s disease, and eosinophilic polyangiitis granulomatous polyangiitis (EPGA). The detection of ANCA by ELISA is essential for the diagnosis of these types of vasculitis. The characteristics of ANCA-related vasculitis in children compared with adults are high fever, more frequent involvement of the nasal cavity and abdominal ischemia, a higher relapse rate, more sequelae (nose, ears, throat), and more prolonged treatment [41].

**Figure 12:**
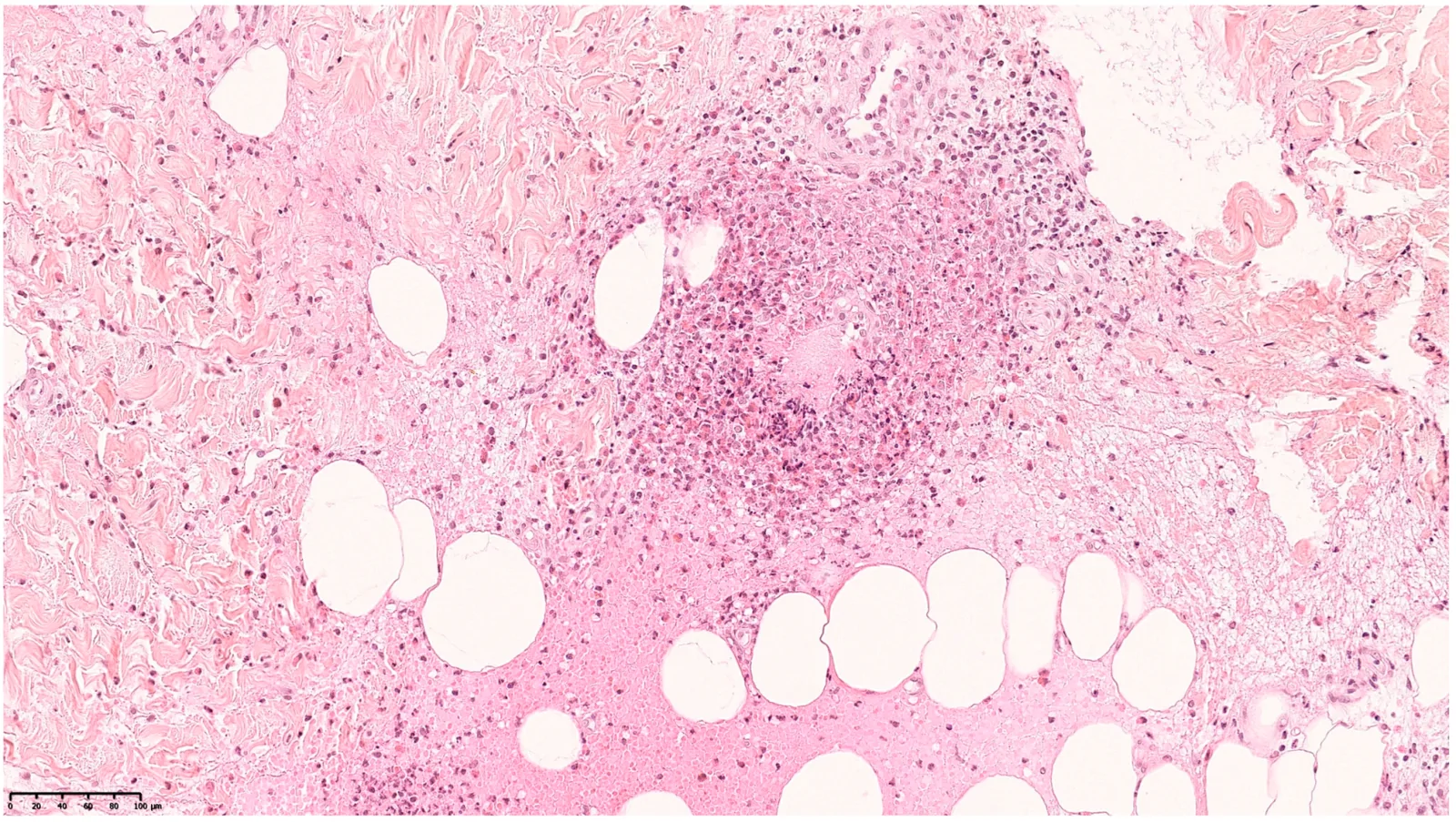
ANCA-related vasculitis, Skin biopsy, hematein eosin, ×40 magnification. Intense mixed inflammatory infiltrates, including neutrophils, lymphocytes, histiocytes, and eosinophils, around a destroyed vessel. This figure is from Necker Pathology department and illustrates the corresponding pathology.

HISTOLOGY (Figure 12).

GPA: Cutaneous lesions in vasculitis often reveal leukocytoclastic vasculitis affecting small- to medium-sized dermal vessels, with fibrinoid necrosis, neutrophilic infiltrates, and red blood cell extravasation. Studies report this pattern in up to 80% of skin biopsies from affected patients, though non-specific chronic inflammation is also common. True granulomatous vasculitis is very rare. Other findings may include extensive necrotic areas and lesions resembling erythema nodosum or granuloma annulare [9,42,43].

EGPA: Cutaneous lesions in eosinophilic granulomatosis with polyangiitis are diverse, often showing extravascular “Churg-Strauss” granulomas. Early lesions present with collagen degeneration and mixed inflammatory infiltrates, including neutrophils, lymphocytes, histiocytes, and eosinophils, sometimes with flame figures [9,43,44].

PAM: Microscopic polyangiitis is marked by small-vessel leukocytoclastic vasculitis, affecting arterioles, capillaries, and venules. It shows necrotizing vasculitis with fibrinoid necrosis and an infiltrate that evolves from neutrophils with karyorrhexis in early stages to lymphocytes and histiocytes in older lesions. Granulomatous inflammation and large-vessel disease are typically absent [9,43,45].

## 9. Behcet Disease

Behçet’s disease is a vasculitis that can involve vessels of all calibers in both the arterial and venous systems. Its incidence is rare in children (between 3% and 26% of all Behçet cases). Pediatric Behçet generally occurs at a median age of 8 years, with delayed diagnosis after an average of 6 years [46].

Classification criteria have been established to aid in the diagnosis of Behçet’s disease in children, with ≥3 of the following signs:

- Recurrent oral aphthosis (≥3 episodes/year);
- Typical genital ulceration or aphthosis with scarring;
- Skin involvement: folliculitis, acneiform lesions, or erythema nodosum;
- Ocular involvement: anterior uveitis, posterior uveitis, or retinal vasculitis;
- Neurological signs, with the exception of isolated headaches;
- Vascular signs: venous thrombosis, arterial thrombosis, or arterial aneurysms.

Skin biopsy is not included among the diagnostic criteria. Its aspect depends on the type of lesion sampled [9].

In most cases, features of vasculitis are observed (up to 50% in one study) [47], including lymphocytic vasculitis and, in some instances, leukocytoclastic vasculitis associated with phlebitis or arteriolitis [48].

Vasculitis is not always present in pathergic lesion which can present with Sweet-syndrome-like aspects [49].

Oral and genital ulcerations often lack specific characteristics but may occasionally exhibit histological signs of leukocytoclastic or lymphocytic vasculitis.

## 10. Takayasu Arteritis

Takayasu arteritis (TAK) is a rare large vessel vasculitis affecting the aorta and its major branches. The mean age at disease onset in the pediatric population is 12 years [50]. Clinical manifestations in children are not very specific: fever, arthromyalgia, weight loss, headache, malaise, and hypertension. The aortic and renal involvement is more frequent in children, often resulting in arterial hypertension [51,52]. There is no vasculitis-like skin involvement.

Treatment is based on corticoids and immunosuppressants or more targeted therapies (TNFα- or IL-6- and JAK-inhibitors).

Skin biopsy is not often performed, but if done, the usual presentation is giant-cell arteritis, with leukocytoclastic and lymphocytic vasculitis and polyarteritis nodosa-like aspects [53,54].

Erythema nodosum lesions show septal and lobular panniculitis with granulomatous vasculitis. Churg–Strauss aspects have also been reported.
**Clinical and Histologic Aspect of Cutaneous Pediatric Vasculitis**
**Clinical Aspects****Histologic Aspects**Common VasculitisIgA VasculitisCutaneous: -Palpable vascular purpura, which may be necrotic, bullous, or crusted. -Predominantly present on the buttocks and lower limbs. Extra Cutaneous: -Arthralgia or arthritis. -Gastrointestinal involvement: abdominal pain, vomiting, rectal bleeding, or diarrhea -Renal involvement: hematuria with or without proteinuria, nephritic and/or nephrotic syndrome. -Other: orchitis, neurological involvement, or pulmonary involvement.-Leukocytoclastic vasculitis. -Often fibrinoid necrosis. -Neutrophilic infiltration. -Important leukocytoclasia sometimes. -Often features extravasation of red blood cells. -Frequently includes eosinophils. -Absence of IgA deposits does not rule out the diagnosis.AHEI-Palpable purpuric lesions, predominantly affecting the face, ears, arms, forearms, thighs, and legs. -Hemorrhagic edema, ecchymotic, annular, and nummular lesions. - Symmetric distribution -fever but good overall health.-Same as IgA vasculitis. -Inflammatory infiltrate more polymorphous (lymphocytes, neutrophils, eosinophils). -Distribution interstitial, perivascular, deep, peri-adnexal.Polyarteritis Nodosa-Fever. -Cutaneous: subcutaneous nodules, livedo, purpura, and skin ulcers. -Extra Cutaneous: arthralgia and myalgia, digestive, neurological, and renal involvement.-Serial cuts are required (vascular involvement segmental and focal). -necrotizing vasculitis of medium-sized vessels, fibrinoid necrosis, leukocytoclasia. -Neutrophilic infiltration around and within arterial walls. -Extravasation of red blood cells. -Possible eosinophils and lymphocytes. -Different stages of development. Kawasaki DiseaseFever persisting for more than 5 days, unresponsive to antipyretics, accompanied by at least four of the following criteria: -Changes in extremities. -Cervical lymphadenopathy greater than 1.5 cm in diameter. -Non-specific maculopapular erythema. -Alterations in lips and oral mucosa. -Bilateral non-purulent conjunctivitis.Skin biopsy not recommended as a diagnostic criterion given its lack of specificity. New EntitiesCOVID-19 Vasculitis-Chilblain-like lesions. -Livedoid and purpuric lesions. -Polymorphic rash. -Perineal and face desquamation. -Hand and feet anomalies (erythema, edema, and induration).-Viral cytopathic changes + secondary inflammatory processes. -Not mandatory and not specific. -Chilblain-like = lymphocytic vasculitis of small vessels + variably fibrinoid necrosis, endothelitis, thrombosis, and extravasation of red blood cells. -COVID-19-induced chilblains indistinguishable from other causes.DADA2Cutaneous: livedo reticularis, cutaneous features resembling polyarteritis nodosa, Raynaud’s phenomenon, and ulcers or necrosis Extra Cutaneous: -Neurological involvement with stroke. -Fever. -Lymphoproliferation: hepatosplenomegaly and lymphadenopathy.-Close to PAN aspect. -Interstitial infiltration by neutrophils around and within small- and medium-sized arteries. -Possible mononuclear cell infiltrate surrounding thrombosed intermediate-sized vessels simulating lymphocytic thrombophilic arteritis.SAVI-Fever. -Skin vasculopathy: telangiectasias, chilblain-like lesions, livedo, violaceous nodules or plaques, ulcerations, and nasal perforation. -Joint inflammation. -Interstitial lung disease.-Thrombotic microangiopathy. -Small-vessel lymphocytic vasculitis. -Neutrophilic dermatosis sometimes.ANCA-related Vasculitis-Cutaneous: purpura. -Extra Cutaneous: fever, more frequent involvement of the nasal cavity, and abdominal ischemia.-GPA: leukocytoclastic vasculitis of small- to medium-sized dermal vessels, fibrinoid necrosis, neutrophilic infiltrates, and red blood cell extravasation. True granulomatous vasculitis is very rare. Possible extensive necrotic areas and lesions resembling erythema nodosum or granuloma annulare. -EGPA: often extravascular “Churg–Strauss” granulomas. Early lesions = degeneration and mixed inflammatory infiltrates, including neutrophils, lymphocytes, histiocytes, and eosinophils, sometimes with flame figures. -PAM: small-vessel leukocytoclastic vasculitis of arterioles, capillaries, and venules, necrotizing vasculitis with fibrinoid necrosis. Infiltrate with neutrophils and karyorrhexis in early stages, lymphocytes, and histiocytes in older lesions. BEHCET DiseaseBehçet’s disease in children, with ≥3 of the following signs: Cutaneous: -Recurrent oral aphthosis (≥3 episodes/year). -Typical genital ulceration or aphthosis with scarring. -Skin involvement: folliculitis, acneiform lesions, or erythema nodosum. Extra Cutaneous: -Ocular involvement. -Neurological signs. -Vascular signs: thrombosis or arterial aneurysms.Skin biopsy is not included in the diagnostic criteria.TAKAYASU Disease-Fever, arthromyalgia, weight loss, headache, malaise, and hypertension. -Aortic and renal involvement. -No vasculitis-like skin involvement.Giant-cell arteritis, with leukocytoclastic and lymphocytic vasculitis and PAN aspect.

## 11. Conclusions

The management of pediatric cutaneous vasculitis requires a precise anatomo-clinical approach, with close collaborations between the dermatologist and the pathologist around the aspects, distribution, and temporal evolution of lesions.

Although skin histology is not always mandatory in the context of vasculitis, it can be crucial in certain diseases, sometimes enabling early diagnosis (e.g., PAN, DADA2) or confirming the diagnosis when clinical features are atypical. Interdisciplinary collaboration among dermatologists, pathologists, and other specialists is essential to ensure accurate diagnosis and effective treatment strategies. Ongoing research and collaborative efforts remain crucial to improve our understanding and management of these complex conditions.

## Data Availability

No new data were created or analyzed in this study. Data sharing is not applicable to this article. Figures included in this article that were not previously published are provided solely for illustrative purposes, to support the explanation of existing concepts. These figures do not constitute primary data and were not used to derive or infer any new conclusions.

## Abbreviations

AHEI	Acute Hemorragic Edema of Infancy
DADA2	Deficiency of adenosine deaminase 2
SAVI	STING-associated vasculopathy with onset in infancy
PAN	Polyarteritis Nodosa
